# Percutaneous Mechanical Aspiration for Right-Sided Infective Endocarditis in People Who Inject Drugs and Those With Cardiac Devices: A Multicenter Analysis

**DOI:** 10.1016/j.jscai.2026.105493

**Published:** 2026-06-30

**Authors:** Kiyan Heybati, Guy Robinson, Benjamin Hibbert, Sripal Bangalore, Pete Fong, David Zlotnick, Bassim El-Sabawi, Brittany Zwischenberger, Ahmad Mourad, Sameh Sayfo, Stephanie Younes, Joseph Ingrassia, Muhammad Hammadah, Anand Prasad, Nadira Hamid, Pedro Villablanca, Amir Kaki, Mohammed Qintar, Marquand Patton, Evin Yucel, Pradeep Yada, Andrew Klein, Larry Baddour, Paul Sorajja, John Moriarty, Sahil A. Parikh, Sanjum Sethi, Abdallah El Sabbagh

**Affiliations:** a Department of Internal Medicine, Mayo Clinic, Rochester, Minnesota; b Department of Internal Medicine, Mayo Clinic, Jacksonville, Florida; c Department of Cardiovascular Disease, Mayo Clinic, Rochester, Minnesota; d Division of Cardiovascular Disease, New York University School of Medicine, New York, New York; e Division of Cardiovascular Disease, Vanderbilt University Medical Center, Nashville, Tennessee; f Division of Cardiovascular Disease, Jacobs School of Medicine and Biomedical Sciences, University at Buffalo, Buffalo, New York; g Division of Cardiothoracic Surgery, Duke University, Durham, North Carolina; h Division of Infectious Diseases, Duke University, Durham, North Carolina; i Division of Cardiovascular Disease, Baylor Scott & White The Heart Hospital − Plano, Plano, Texas; j Division of Cardiovascular Medicine, Promedica Toledo, Toledo, Ohio; k Division of Cardiovascular Disease, Hartford Healthcare Heart and Vascular Institute, Hartford, Connecticut; l Division of Cardiovascular Disease, University of Texas Health San Antonio, San Antonio, Texas; m Department of Cardiovascular Disease, Minneapolis Heart Institute, Minneapolis, Minnesota; n Division of Cardiovascular Disease, Henry Ford Hospital, Detroit, Michigan; o Division of Cardiovascular Disease, Ascension Michigan St. John Hospital, Detroit, Michigan; p Division of Cardiovascular Disease, Sparrow Medical Center, Lansing, Michigan; q Division of Cardiovascular Disease, Palmetto General Hospital, Hialeah, Florida; r Division of Cardiovascular Disease, Massachusetts General Hospital, Boston, Massachusetts; s Division of Cardiovascular Disease, Piedmont Atlanta Hospital, Atlanta, Georgia; t Division of Public Health, Infectious Diseases and Occupational Medicine, Department of Medicine, Mayo Clinic College of Medicine and Science, Rochester, Minnesota; u Department of Radiology and Department of Cardiology, University of California, Los Angeles, Los Angeles, California; v Division of Cardiovascular Disease, NewYork-Presbyterian Hospital/Columbia University Irving Medical Center, New York, New York; w Department of Cardiovascular Medicine, Mayo Clinic, Jacksonville, Florida

**Keywords:** aspiration, cardiac devices, drug use, endocarditis, patient outcomes

Right-sided infective endocarditis (RSIE) is increasingly prevalent, driven by injection drug use and utilization of cardiac implantable electronic devices (CIED).^1^^,^^2^ Percutaneous mechanical aspiration (PMA) has emerged as a catheter-based strategy for vegetation debulking in RSIE.^3^ In the Cardiac Lesion Extraction and Aspiration Registry for Infective Endocarditis (CLEAR-IE), PMA demonstrated high procedural success, with adverse events primarily reflecting underlying disease severity.^3^ However, the cohort was heterogeneous, with RSIE most commonly occurring in people who inject drugs (PWID) and patients with CIED-related infections. This subanalysis compared characteristics and outcomes of PMA in both populations with RSIE.

CLEAR-IE is a multicenter registry from 19 US centers evaluating outcomes of PMA in RSIE; its design has been described previously.^3^ Eligible patients met modified Duke criteria for IE and underwent PMA at the discretion of the treating team. Baseline characteristics, procedural details, and outcomes were retrospectively collected using a standardized form, with ethical approval at all sites. Reporting followed STROBE guidelines (Supplemental Table S1).^4^

We included consecutive RSIE patients treated with PMA with a history of injection drug use or CIED infection; patients with left-sided endocarditis were excluded. Patients were stratified into PWID or CIED groups (Supplemental Figure S1). To ensure mutually exclusive groups, PWIDs with underlying CIED were excluded. The primary outcome was procedural success, defined as ≥70% reduction in vegetation size or residual vegetation ≤10 mm. Secondary outcomes included a composite of adverse events (in-hospital death, new pulmonary embolism, worsening tricuspid regurgitation, or emergency surgery) and each component individually, along with cardiac surgery within 6 weeks, as well as 6-week survival.

Statistical analyses were performed in R version 4.4.2 (R Foundation for Statistical Computing). Descriptive statistics were used with complete case analysis due to the limited amount of missing data (Supplemental Table S2). Associations between CIED or PWID status and outcomes were evaluated using multivariable logistic regression, adjusting for prespecified covariates based on clinical expertise and current literature (age at time of procedure, patient sex, *Staphylococcus* infection, prior IE, prior MI, hypoxia, acute pulmonary embolism, and prior pulmonary hypertension and/or COPD requiring oxygen; Table 1, Supplemental Table S2). Collinearity was assessed using variance inflation factors. Results are reported as odds ratios (OR) with 95% CI. To limit overfitting, model complexity was constrained to 1 covariate per 10 outcome events. Survival was evaluated using Kaplan–Meier methods and adjusted Cox proportional hazards models, reported as adjusted hazard ratios (HR) with 95% CI. A 2-sided *P* value < .05 denoted statistical significance.

**Table 1:** Patient characteristics, procedural characteristics, and clinical outcomes stratified by patient type

Variable	PWID (n = 122)	CIED (n = 60)	*P* value
Patient characteristics			
Age at time of procedure, y	36.0 (33.0-41.0)	65.0 (59.0-73.0)	<.001^e^
Female sex	57 (46.7)	20 (33.3)	.086
HTN	5 (4.1)	44 (73.3)	<.001^e^
Hyperlipidemia	0 (0.0)	32 (53.3)	<.001^e^
Diabetes mellitus	1 (0.8)	29 (48.3)	<.001^e^
Myocardial infarction	1 (0.8)	16 (26.7)	<.001^e^
Stroke	3 (2.5)	7 (11.7)	.010^e^
Pulmonary HTN/COPD on O_2_	2 (1.6)	17 (28.8)	<.001^e^
Atrial fibrillation/flutter	0 (0.0)	30 (50.8)	<.001^e^
Dialysis	3 (2.5)	6 (10.0)	.027^e^
Prior endocarditis	33 (28.7)	3 (5.8)	<.001^e^
Prior percutaneous coronary intervention	0 (0.0)	21 (35.0)	<.001^e^
Prior CABG	0 (0.0)	13 (21.7)	<.001^e^
Left ventricular ejection fraction, %	55.0 (55.0-60.0)	49.0 (31.3-55.0)	<.001^e^
Procedural indications/rationale (≥1/patient)			
Large vegetation	105 (86.1)	27 (45.0)	<.001^e^
Ongoing sepsis	95 (77.9)	25 (41.7)	<.001^e^
Recurrent embolism despite therapy	31 (25.6)	2 (3.3)	<.001^e^
Other	23 (18.9)	11 (18.3)	.93
Hospitalization/procedure characteristics			
Hypoxia	50 (41.0)	15 (25.0)	.034^e^
Acute pulmonary embolism	98 (80.3)	7 (11.7)	<.001^e^
Acute heart failure	11 (9.0)	16 (26.7)	.002^e^
Positive blood cultures	118 (96.7)	52 (86.7)	.010^e^
Vegetation size, long, mm	23.0 (19.0-28.0)	26.5 (19.8-35.0)	.35
Staphylococcus species^a^	106 (88.3)	29 (49.2)	<.001^e^
Continuous suction device	107 (93.9)	37 (63.8)	<.001^e^
Procedure time, min	88.0 (64.5-125.0)	105.5 (87.0-140.5)	.007^e^
Select outcomes			
Composite success	91 (84.3)	55 (96.5)	.019^e^
Composite adverse events	40 (32.8)	18 (30.0)	.70
In-hospital death	8 (6.6)	9 (15.0)	.066
New pulmonary embolism	11 (9.0)	4 (6.8)	.61
Worsening tricuspid regurgitation	24 (20.9)	8 (15.1)	.38
Emergency surgery	4 (3.3)	2 (5.1)	.56
Residual vegetation	95 (77.9)	22 (37.3)	<.001^e^
Hospital length of stay, d	23.0 (13.0-47.3)	15.0 (11.0-23.3)	<.001^e^
Blood culture clearance	109 (90.8)	50 (92.6)	.70
Cardiac surgery within 6 wk	5 (5.7)	3 (6.8)	.80
Adjusted outcomes			
Procedural success, OR (95% CI)^b^	0.16 (0.01-1.85)	Reference	.17
Adverse events, OR (95% CI)^c^	23.07 (4.58-139.60)	Reference	<.001^e^
6-Week survival, HR (95% CI)^d^	2.62 (0.42-16.2)	Reference	.30

Values are presented as n (%) or median (IQR), unless otherwise specified. Missing variable data are presented in Supplemental Table S2. All other variables had complete data.

CABG, coronary artery bypass graft; CIED, cardiac implantable electronic device; COPD, chronic obstructive pulmonary disease; HR, hazard ratio; HTN, hypertension; PWID, people who inject drugs; OR, odds ratio.

a *S. aureus*, *S. lugdunensis*, other coagulase-negative staphylococci.

b Adjusted for age at time of procedure, patient sex, Staphylococcus infection, prior history of endocarditis, and prior MI. Total N = 147.

c Adjusted for age at time of procedure, Staphylococcus infection, hypoxia, and acute pulmonary embolism. Total N = 178.

d Adjusted for age at time of procedure, patient sex, Staphylococcus infection, prior history of endocarditis, prior MI, and prior pulmonary HTN/COPD on O_2_. Total N = 151.

e Significant finding, *P* < .05.

Among 182 patients with RSIE who underwent PMA, 122 (67.0%) were PWIDs and 60 (33.0%) had CIED-associated IE (Table 1; Supplemental Table S2). Median age was 42.0 years (IQR, 35.0-61.0), and 77 patients (42.3%) were female. PWIDs were younger than patients with CIED (36.0 [33.0-41.0] vs 65.0 [59.0-73.0] years; *P* < .001) and frequently had prior IE (28.7% vs 5.8%; *P* < .001), whereas patients with CIED had greater baseline comorbidity burden. PWIDs often presented with pulmonary embolism (80.3% vs 11.7%; *P* < .001), hypoxia (41.0% vs 25.0%; *P* = .034), and persistent sepsis despite antimicrobial treatment (77.9% vs 41.7%; *P* < .001), whereas acute heart failure was more frequent among patients with CIED. *Staphylococcus* species were more common in PWIDs (88.3% vs 49.2%; *P* < .001). Baseline vegetation size was similar (PWID, 23.0 [19.0-28.0] vs CIED, 26.5 [19.8-35.0] mm; *P* = .35). General anesthesia use was comparable (PWID, 86.7% vs CIED, 90.2%; *P* = .48); however, procedural duration was longer in the CIED group (105.5 [87.0-140.5] vs 88.0 [64.5-125.0] minutes). Continuous-flow aspiration was more frequently employed in PWIDs (93.9% vs 63.8%; *P* < .001), and residual vegetation was more common (77.9% vs 37.3%; *P* < .001) but similar in size (PWID, 0.73 [0.40-1.18] vs CIED, 0.50 [0.00-0.98] mm; *P* = .39).

People who inject drugs had lower unadjusted odds of procedural success (OR, 0.19; 95% CI, 0.03-0.71; *P* = .033); however, this difference was no longer significant after adjustment for age, sex, Staphylococcus infection, prior IE, and prior myocardial infarction (adjusted OR, 0.16; 95% CI, 0.01-1.85; *P* = 0.17; *N* = 147). Although unadjusted odds of composite adverse events were similar (OR, 1.14; 95% CI, 0.59-2.25; *P* = .70), PWIDs demonstrated significantly higher adjusted odds of adverse events compared with that of the CIED group (adjusted OR, 23.07; 95% CI, 4.58-139.60; *P* < .001; *N* = 178). Rates of worsening tricuspid regurgitation, emergency surgery, and new pulmonary embolism were similar (Table 1). PWID experienced longer hospital stays (23.0 [13.0-47.3] vs 15.0 [11.0-23.3] days; *P* < .001) and higher rates of self-directed discharge (15.8% vs 0.0%; *P* = .003). Post-procedural blood culture clearance exceeded 90% in both groups and did not differ significantly. There was no significant difference in adjusted survival between PWID and CIED cohorts at 6 weeks (adjusted HR, 2.62; 95% CI, 0.42-16.2; *P* = .30; *N* = 151; Supplemental Figure S2). There were no significant differences in the proportion of patients undergoing cardiac surgery within 6 weeks (*P* = .80; Table 1).

This subanalysis demonstrates that PWID and patients with CIED represent distinct RSIE phenotypes and should be evaluated as such in clinical investigations. Consistent with prior reports, PWID were younger and had fewer comorbidities yet presented with more advanced RSIE, whereas CIED patients were older with greater comorbidity burden.^2^^,^^5^^,^^6^

Procedural success was high in both groups, aligning with prior series.^3^ In CIED-related infections, PMA is frequently used to facilitate lead extraction by reducing vegetation dislodgement risk with subsequent pulmonary complications, which may explain longer procedure times and lower use of continuous aspiration (eg, AngioVac), an approach that requires large-bore access, perfusion circuit, and specialized team. Despite the perceived higher procedural complexity in CIED infections, adjusted adverse events were more common in PWID, likely reflecting their more aggressive disease phenotype caused by a higher prevalence of *Staphylococcus aureus* as a pathogen, including septic emboli, persistent sepsis, and hypoxia. Additional granular pathological data are also needed to provide more insights into the characteristics of the valvular vegetations such as the degree of fibrosis. The PWID group also exhibited high rates of self-directed discharge, consistent with prior literature.^1^^,^^7^^,^^8^ Although not statistically significant, in-hospital mortality was numerically higher in the CIED group, which may be reflective of the older population with more comorbid conditions and the complexity of potential lead extraction.

The retrospective design and modest sample size introduce risks of data misclassification, residual confounding, and reporting bias. Reliance on site-reported data without core laboratory adjudication or standardized protocols may have contributed to variability. Comparisons between two clinically distinct groups (ie, PWID and CIED) also introduce inherent biases, as differences in demographics and care may influence outcomes beyond what is captured analytically. Sample size limitations may have impacted the ability to identify potentially significant differences between groups, as well as contributing to wide confidence intervals. The absence of uniform eligibility criteria further limits interpretability.

Overall, these findings underscore the need for prospective studies that stratify patients into clinically distinct RSIE cohorts to more accurately define safety, efficacy, and patient selection for PMA. Studies should account for cohort-specific factors such as substance use cessation, concomitant procedures (eg, lead extraction), and challenges related to follow-up.

## CRediT authorship contribution statement

**Kiyan Heybati:** Writing – review & editing, Writing – original draft, Visualization, Validation, Software, Methodology, Investigation, Formal analysis, Data curation, Conceptualization. **Guy Robinson:** Writing – review & editing, Visualization, Validation, Methodology, Data curation. **Benjamin Hibbert:** Writing – review & editing, Visualization, Validation, Methodology, Investigation, Data curation, Conceptualization. **Sripal Bangalore:** Writing – review & editing, Validation, Methodology, Investigation, Data curation, Conceptualization. **Pete Fong:** Writing – review & editing, Validation, Methodology, Investigation, Data curation, Conceptualization. **David Zlotnick:** Writing – review & editing, Methodology, Investigation, Data curation, Conceptualization. **Bassim El-Sabawi:** Writing – review & editing, Validation, Methodology, Investigation, Data curation, Conceptualization. **Brittany Zwischenberger:** Writing – review & editing, Validation, Methodology, Investigation, Data curation, Conceptualization. **Ahmad Mourad:** Writing – review & editing, Validation, Methodology, Investigation, Data curation, Conceptualization. **Sameh Sayfo:** Writing – review & editing, Validation, Investigation, Data curation, Conceptualization. **Stephanie Younes:** Writing – review & editing, Validation, Methodology, Investigation, Data curation, Conceptualization. **Joseph Ingrassia:** Writing – review & editing, Validation, Methodology, Data curation, Conceptualization. **Muhammad Hammadah:** Writing – review & editing, Validation, Investigation, Data curation, Conceptualization. **Anand Prasad:** Writing – review & editing, Validation, Methodology, Data curation, Conceptualization. **Nadira Hamid:** Writing – review & editing, Validation, Methodology, Investigation, Data curation, Conceptualization. **Pedro Villablanca:** Writing – review & editing, Validation, Methodology, Investigation, Data curation, Conceptualization. **Amir Kaki:** Writing – review & editing, Validation, Methodology, Investigation, Data curation, Conceptualization. **Mohammed Qintar:** Writing – review & editing, Validation, Methodology, Investigation, Data curation, Conceptualization. **Marquand Patton:** Writing – review & editing, Validation, Methodology, Investigation, Data curation, Conceptualization. **Evin Yucel:** Writing – review & editing, Validation, Methodology, Investigation, Data curation, Conceptualization. **Pradeep Yada:** Writing – review & editing, Validation, Methodology, Investigation, Data curation, Conceptualization. **Andrew Klein:** Writing – review & editing, Validation, Methodology, Investigation, Data curation, Conceptualization. **Larry Baddour:** Writing – review & editing, Validation, Methodology, Investigation, Data curation, Conceptualization. **Paul Sorajja:** Writing – review & editing, Validation, Investigation, Data curation, Conceptualization. **John Moriarty:** Writing – review & editing, Validation, Investigation, Data curation, Conceptualization. **Sahil A. Parikh:** Writing – review & editing, Methodology, Investigation, Data curation, Conceptualization. **Sanjum Sethi:** Writing – review & editing, Validation, Supervision, Project administration, Methodology, Investigation, Funding acquisition, Data curation, Conceptualization. **Abdallah El Sabbagh:** Writing – review & editing, Writing – original draft, Validation, Supervision, Resources, Project administration, Methodology, Investigation, Funding acquisition, Data curation, Conceptualization.

## Peer review statement

Associate Editor Sahil A. Parikh had no involvement in the peer review of this article and has no access to information regarding its peer review. Full responsibility for the editorial process for this article was delegated to Deputy Editor Suzanne J. Baron.

## Declaration of competing interest

Sripal Bangalore serves on advisory boards for Abbott Vascular, Boston Scientific, Biotronik, Amgen, Pfizer, Merck, Reata, Inari, and Truvic. David Zlotnick serves on speakers’ bureaus for Abiomed, Inari Medical, and AngioDynamics and is a consultant for AngioDynamics. Sameh Sayfo is a consultant for AngioDynamics, Inari Medical, Penumbra, Imperative Care, and Boston Scientific. Stephanie Younes serves on the speakers’ bureau for AngioDynamics. Nadira Hamid consults for Abbott Structural, Anteris, AMX, 4C Medical Technologies, Alleviant Medical, Edwards Lifesciences, Philips, GE, Valcare Med Ltd, vDyne, and W. L. Gore. Pedro Villablanca is a consultant for Edwards Lifesciences, Medtronic, AngioDynamics, and Abiomed. Marquand Patton is a consultant for AngioDynamics. Paul Sorajja consults for 4C Medical, Abbott Structural, Adona, Boston Scientific, ConKay, CroiValve, Cultiv8, Edwards Lifesciences, Egg Medical, EvolutionMed, Foldax, GE Medical, Haemonetics, InQ8, LAZA, Medtronic, Philips, Polares, W. L. Gore, vDyne, Unorthodox Ventures, Valcare, and xDot and has received institutional research grants from Abbott, Boston Scientific, Edwards Lifesciences, and Medtronic. John Moriarty has received support for the current manuscript from Penumbra; has received consulting fees from AngioDynamics, Innova Vascular, Inquis Medical, Boston Scientific, Imperative Care, and Thrombolex; has received payment or honoraria for lectures, presentations, speakers’ bureaus, manuscript writing, or educational events from AngioDynamics and Penumbra; and has stock or stock options from PAVmed and Thrombolex. Sahil Parikh has received institutional grants and research support from Abbott Vascular, Shockwave Medical, TriReme Medical, SurModics, Silk Road Medical, and the National Institutes of Health; has received consulting fees from Terumo and Abiomed; and has served on the advisory boards of Abbott, Medtronic, Boston Scientific, CSI, Janssen, and Philips. Sanjum Sethi has received honoraria from Boston Scientific, Terumo, Inari, Penumbra, Janssen, and Chiesi. Kiyan Heybati, Guy Robinson, Benjamin Hibbert, Pete Fong, Bassim El-Sabawi, Brittany Zwischenberger, Ahmad Mourad, Joseph Ingrassia, Muhammad Hammadah, Anand Prasad, Amir Kaki, Mohammed Qintar, Evin Yucel, Pradeep Yada, Andrew Klein, Larry Baddour, and Abdallah El Sabbagh reported no financial interests.
