# Supplementary material for: Percutaneous Mechanical Aspiration for Right-Sided Infective Endocarditis in People Who Inject Drugs and Those With Cardiac Devices: A Multicenter Analysis
Source: J Soc Cardiovasc Angiogr Interv. 2026 Jun 30;5(8):105493. doi: 10.1016/j.jscai.2026.105493 (PMC13491325; doi:10.1016/j.jscai.2026.105493)
Supplement: Supplementary file 1 [file mmc1.docx]

**ELECTRONIC SUPPLEMENTARY MATERIAL**

**Percutaneous Mechanical Aspiration for Right-Sided Infective Endocarditis in People Who Inject Drugs and Those with Cardiac Devices: A Multi-Center Analysis**

Kiyan Heybati^1^, MD, MSc; Guy Robinson^2^, MBBS; Benjamin Hibbert ^3^, MD, PhD; Sripal Bangalore^4^, MD, MHA; Pete Fong^5^, MD; David Zlotnick^6^, MD; Bassim El-Sabawi^5^, MD; Brittany Zwischenberger^7^, MD; Ahmad Mourad^8^, MD, MHS; Sameh Sayfo^9^, MD, MBA; Stephanie Younes^10^, MD; Joseph Ingrassia^11^, MD; Muhammad Hammadah^12^, MD; Anand Prasad^13^, MD; Nadira Hamid^13^, MD; Pedro Villablanca^14^, MD, MSc; Amir Kaki^15^, MD; Mohammed Qintar^16^, MD, MSc; Marquand Patton^17^, DO; Evin Yucel^18^, MD; Pradeep Yadav^19^, MD; Andrew Klein^19^, MD; Larry Baddour^20^, MD; Paul Sorajja^13^, MD; John Moriarty^21^, MD; Sahil Parikh^22^, MD; Sanjum Sethi^22^, MD, MPH; Abdallah El Sabbagh^23^, MD

**Table of Contents**

**eTable1.** The Strengthening the Reporting of Observational Studies in Epidemiology (STROBE) checklist………………………………………………………………………………,……..…….……..2-4

**eFigure 1.** Study sites and patient flow diagram…….…………………………………………….….……5

**eTable 2.** Number of missing data, stratified by patient type………………………………………………6

**eFigure 2.** Kaplan-Meier curve demonstrating relative 6-week survival among patients with IV drug use versus cardiac devices………………………………………………………………………………………7

**eTable 1.** The Strengthening the Reporting of Observational Studies in Epidemiology (STROBE) checklist

|  | **Item No.** | **STROBE items** | **Location in manuscript where items are reported** |
| --- | --- | --- | --- |
| Title/abstract | 1 | (a) Indicate the study’s design with a commonly used term in the title or the abstract (b) Provide in the abstract an informative and balanced summary of what was done and what was found | Title page |
| Background/ rationale | 2 | Explain the scientific background and rationale for the investigation being reported | Introduction, Page 3 |
| Objectives | 3 | State specific objectives, including any prespecified hypotheses | Introduction, Page 3 |
| Study Design | 4 | Present key elements of study design early in the paper | Methods, Pages 3 |
| Setting | 5 | Describe the setting, locations, and relevant dates, including periods of recruitment, exposure, follow-up, and data collection | Methods, Pages 3 |
| Participants | 6 | *(a) Cohort study* - Give the eligibility criteria, and the sources and methods of selection of participants. Describe methods of follow-up  *Case-control study* - Give the eligibility criteria, and the sources and methods of case ascertainment and control selection. Give the rationale for the choice of cases and controls  *Cross-sectional study* - Give the eligibility criteria, and the sources and methods of selection of participants  *(b) Cohort study* - For matched studies, give matching criteria and number of exposed and unexposed  *Case-control study* - For matched studies, give matching criteria and the number of controls per case | Methods, Pages 3; eFigure 1 |
| Variables | 7 | Clearly define all outcomes, exposures, predictors, potential confounders, and effect modifiers. Give diagnostic criteria, if applicable. | Methods, Pages 3 |
| Data sources/ measurement | 8 | For each variable of interest, give sources of data and details of methods of assessment (measurement).  Describe comparability of assessment methods if there is more than one group | Methods, Pages 3 |
| Bias | 9 | Describe any efforts to address potential sources of bias | Methods, Pages 3 |
| Study size | 10 | Explain how the study size was arrived at | Methods, Pages 3; eFigure 1 |
| Quantitative variables | 11 | Explain how quantitative variables were handled in the analyses. If applicable, describe which groupings were chosen, and why | Methods, Page 3 |
| Statistical methods | 12 | (a) Describe all statistical methods, including those used to control for confounding  (b) Describe any methods used to examine subgroups and interactions  (c) Explain how missing data were addressed  (d) *Cohort study* - If applicable, explain how loss to follow-up was addressed  *Case-control study* - If applicable, explain how matching of cases and controls was addressed  *Cross-sectional study* - If applicable, describe analytical methods taking account of sampling strategy  (e) Describe any sensitivity analyses | Methods, Pages 3 |
| Participants | 13 | (a) Report the numbers of individuals at each stage of the study (*e.g.*, numbers potentially eligible, examined for eligibility, confirmed eligible, included in the study, completing follow-up, and analysed)  (b) Give reasons for non-participation at each stage.  (c) Consider use of a flow diagram | Results, Pages 4; eFigure 1 |
| Descriptive data | 14 | (a) Give characteristics of study participants (*e.g.*, demographic, clinical, social) and information on exposures and potential confounders  (b) Indicate the number of participants with missing data for each variable of interest  (c) *Cohort study* - summarise follow-up time (*e.g.*, average and total amount) | Results, Page 4; Main Table; Main Figure |
| Outcome data | 15 | *Cohort study* - Report numbers of outcome events or summary measures over time  *Case-control study* - Report numbers in each exposure category, or summary measures of exposure  *Cross-sectional study* - Report numbers of outcome events or summary measures | Results, Page 4; Main Table; Main Figure; eFigure 1 |
| Main results | 16 | (a) Give unadjusted estimates and, if applicable, confounder-adjusted estimates and their precision (e.g., 95% confidence interval). Make clear which confounders were adjusted for and why they were included  (b) Report category boundaries when continuous variables were categorized  (c) If relevant, consider translating estimates of relative risk into absolute risk for a meaningful time period | Results, Page 4; Main Table; Main Figure |
| Other analyses | 17 | Report other analyses done—e.g., analyses of subgroups and interactions, and sensitivity analyses | Results, Page 4; Main Table; Main Figure |
| Key results | 18 | Summarise key results with reference to study objectives | Discussion, Pages 4-5 |
| Limitations | 19 | Discuss limitations of the study, taking into account sources of potential bias or imprecision. Discuss both direction and magnitude of any potential bias | Discussion, Pages 4-5 |
| Funding | 22 | Give the source of funding and the role of the funders for the present study and, if applicable, for the original study on which the present article is based | Title page |

**eFigure 1.** Study sites and patient flow diagram

**Panel A.** Map of the United States demonstrating the 19 included study sites. **Panel B.** Patient flow diagram

**Abbreviations:** PMA – percutaneous mechanical aspiration; PWID – people who inject drugs; CIED – cardiac implantable electronic devices.

**eTable 2.** Number of missing data, stratified by patient type

| **Variable** | **PWID (N=122)** | **CIED (N=60)** |
| --- | --- | --- |
| **Age at time of procedure** | 0 | 1 |
| **DM** | 0 | 1 |
| **Pulmonary HTN/COPD on O**₂ | 0 | 1 |
| **Atrial fibrillation/flutter** | 0 | 1 |
| **Prior endocarditis** | 7 | 8 |
| **LVEF** | 5 | 10 |
| **Recurrent embolism despite therapy** | 1 | 0 |
| **Vegetation size, long** | 33 | 20 |
| **Staphylococcus species** | 2 | 1 |
| **Continuous suction device** | 8 | 2 |
| **Procedure time** | 11 | 12 |
| **Composite success** | 14 | 3 |
| **New pulmonary embolism** | 0 | 1 |
| **Worsening tricuspid regurgitation** | 7 | 7 |
| **Emergency surgery** | 1 | 1 |
| **Cardiac surgery within 6 weeks** | 34 | 16 |
| **6-week survival** | 25 | 6 |

Data presented as N. All other variables presented in main Table 1, apart from those in this table, had complete data.

**Abbreviations**: **PWID** – people who inject drugs; **CIED** – cardiac implantable electronic device; **HTN –** hypertension; **DM** – diabetes mellitus; **COPD** – chronic obstructive pulmonary disease; **O_2_** - oxygen; **LVEF** – left ventricular ejection fraction

**eFigure 2.** Kaplan-Meier curve demonstrating relative 6-week survival among patients with IV drug use (i.e. PWID) versus cardiac devices (i.e. CIED)
